# Minimalistic chronic total occlusion treatments using tip detection-antegrade dissection and re-entry: a case series

**DOI:** 10.1093/ehjcr/ytaf355

**Published:** 2025-07-24

**Authors:** Yutaka Tadano, Bayushi Eka Putra, Takuro Sugie, Shoichi Kuramitsu, Umihiko Kaneko, Daitaro Kanno, Tsutomu Fujita

**Affiliations:** Department of Cardiology, Sapporo Cardio Vascular Clinic, 8-1, North 49-East 16, Higashi ward, Sapporo, Hokkaido 007-0849, Japan; Department of Cardiology, Sapporo Cardio Vascular Clinic, 8-1, North 49-East 16, Higashi ward, Sapporo, Hokkaido 007-0849, Japan; Department of Cardiology, Sapporo Cardio Vascular Clinic, 8-1, North 49-East 16, Higashi ward, Sapporo, Hokkaido 007-0849, Japan; Department of Cardiology, Sapporo Cardio Vascular Clinic, 8-1, North 49-East 16, Higashi ward, Sapporo, Hokkaido 007-0849, Japan; Department of Cardiology, Sapporo Cardio Vascular Clinic, 8-1, North 49-East 16, Higashi ward, Sapporo, Hokkaido 007-0849, Japan; Department of Cardiology, Sapporo Cardio Vascular Clinic, 8-1, North 49-East 16, Higashi ward, Sapporo, Hokkaido 007-0849, Japan; Department of Cardiology, Sapporo Cardio Vascular Clinic, 8-1, North 49-East 16, Higashi ward, Sapporo, Hokkaido 007-0849, Japan

**Keywords:** Intravascular ultrasound, Patient safety, Coronary artery disease, Case series, CTO, MAT

## Abstract

**Background:**

Since chronic total occlusion (CTO) percutaneous coronary intervention (PCI) is mainly aimed at symptom relief, it should avoid placing patients at risk. Tip detection-antegrade dissection and re-entry (TD-ADR), whose wiring time has been reported to be shorter compared to retrograde approach, do not need a dual access. Although the retrograde approach is effective, it is a significant risk factor for in-hospital adverse events after CTO PCI. We are advocating ‘Minimalistic Approach with Tip detection-antegrade dissection and re-entry’ (MAT) which positions antegrade wiring as the first, tip-detection intravascular ultrasound-guided wiring as the second, and the retrograde approach as the third option.

**Case summary:**

This case series describes three PCIs performed via a single radial access for right coronary artery CTOs characterized by a puncturable proximal cap and non-diffuse calcification. It was possible for TD-ADR to stick to the CTO body and preserves side branches distal to the CTO.

**Discussion:**

MAT enables antegrade CTO treatment with a single radial access. While TD-ADR is difficult in diffusely calcified CTO, complications of TD-ADR are limited to those that can be adequately anticipated and managed, unlike those associated with the retrograde approach. MAT likely reduces procedural risks and improves patient comfort.

Learning points

- Chronic total occlusion treatment primarily targets symptom relief; therefore, it should not expose patients to excessive risk.
- ‘Minimalistic Approach with Tip detection-antegrade dissection and re-entry’ (MAT) has the potential to not only reduce procedural risks but also lessen the overall burden on patients, including financial costs.

## Introduction

The ‘Minimalistic Hybrid Algorithm’ proposed by Agostoni *et al*.^1^ suggests eliminating the need for 8 Fr sheaths in chronic total occlusion (CTO) percutaneous coronary intervention (PCI). However, their usage rate of retrograde approaches and dual access is still over 30%.

The success rate of tip detection-antegrade dissection and re-entry (TD-ADR) has been reported to reduce wiring time compared to the retrograde approach, and helps minimize retrograde use and dual access in CTO PCIs.^2^ Tip detection-antegrade dissection and re-entry is feasible using a 7 Fr guiding catheter. Although performing TD-ADR can be extremely challenging in cases of diffusely calcified CTOs or when the false lumen is filled with microbubbles, associated complications are generally minor and limited to guidewire exit or distal haematoma extension. Tip detection-antegrade dissection and re-entry is feasible even when distal contrast filling is lost, and unlike Stingray-ADR, it does not require dual access. In general, for right coronary artery (RCA) CTO with a bifurcation distally, the retrograde approach is recommended over Stingray-ADR.^3^ However, it’s possible for TD-ADR to preserve a side branch at the distal CTO cap.^4^

This case series presents CTO PCIs for RCAs via single radial access under a minimalistic concept utilizing the support of TD-ADR.

## Summary figure

**Figure:**
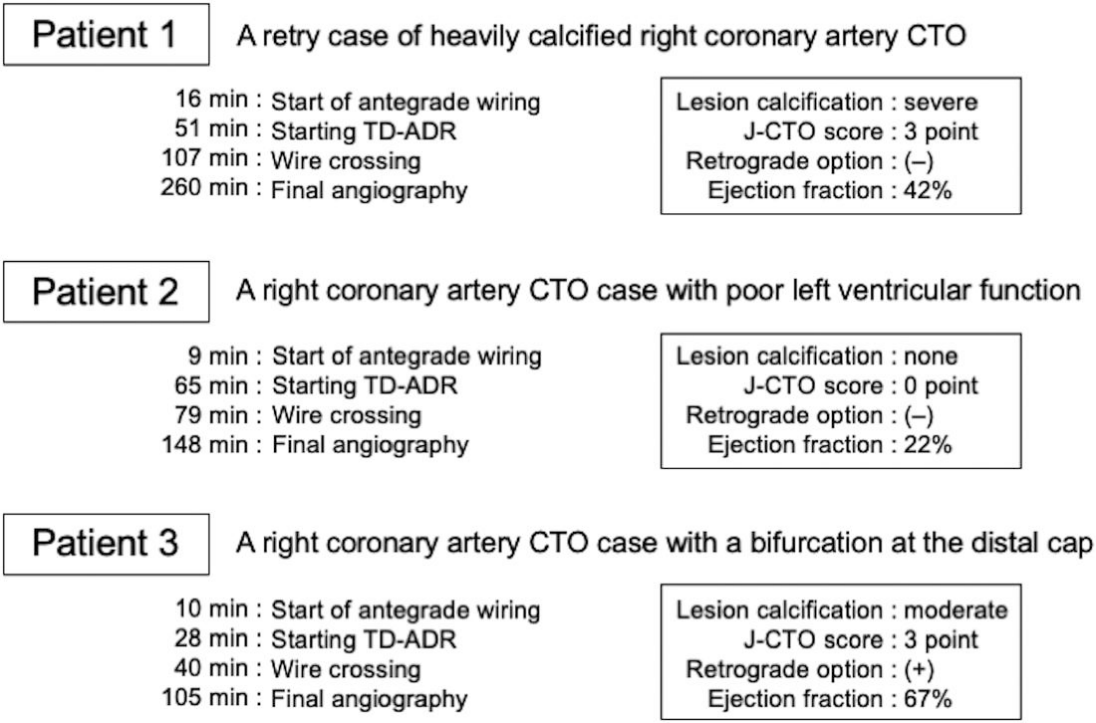


## Case presentation

### Patient 1

**Figure 1:**
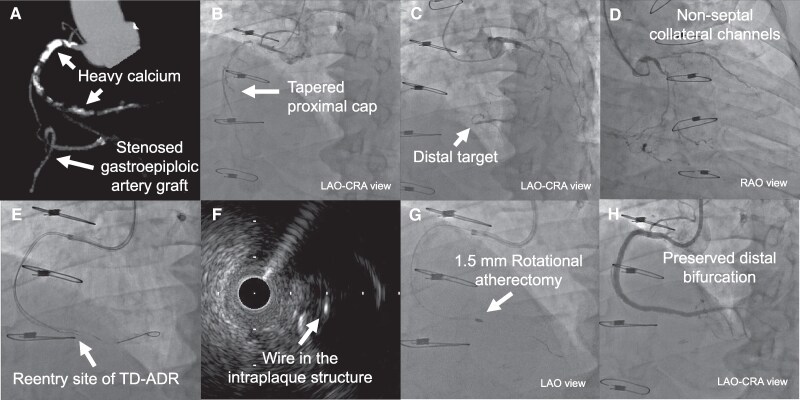
Images of patient 1: (*A*) CT angiogram showing heavy calcification and a stenosed gastroepiploic artery graft. (*B*) Right coronary angiography demonstrating a tapered proximal cap. (*C*) Left coronary angiography highlighting the distal target. (*D*) Left coronary angiography showing non-septal collateral channels. (*E*) Re-entry site of tip detection-antegrade dissection and re-entry (TD-ADR). (*F*) A Conquest Pro 12ST guidewire in the intraplaque structure. (*G*) Rotational atherectomy using a 1.5 mm burr for the distal uncrossable lesion. (*H*) Final angiogram showing a preserved distal bifurcation.

A 70-year-old Japanese man with diabetes and normal renal function presented with persistent exertional chest pain and reduced exercise tolerance despite maximal medical therapy. Electrocardiogram showed ST depression in leads I and V6, and echocardiography revealed diffuse left ventricular hypokinesis (ejection fraction 42%). He had coronary artery bypass grafting at age 48, with patent internal mammary artery grafts to the left anterior descending artery and left circumflex artery, but severe stenosis in the posterior descending artery (PD) gastroepiploic artery graft (*Figure 1A*). He underwent repeat PCI for an RCA CTO (*Figure 1B–D*, see Supplementary material online, *Videos S1*–*S3*). The prior attempt, made four months earlier, was unsuccessful due to severe calcification. No posterolateral branch (PL)-PD communication was noted. J-CTO score was 3, PROGRESS CTO score 1. Via a left radial access using 7 Fr Glidesheath Slender (TERUMO, Japan), we advanced a wire into the extraplaque space and performed TD-ADR through Hyperion (ASAHI INTECC, Japan) 7 Fr AL1 SH guiding catheter (*Figure 1E* and *F*). Severe calcification made wire visualization difficult. By selecting a less calcified puncture site and repeatedly attempting TD-ADR using Conquest Pro 12ST (ASAHI INTECC, Japan) wire, Finecross GT (TERUMO, Japan) microcatheter, and AnteOwl (TERUMO, Japan) intravascular ultrasound (IVUS) catheter, we achieved successful crossing. After predilatation, balloon indentation persisted at segments 1 and 3, and IVUS catheter could not pass segment 3 (*Figure 1G*, see Supplementary material online, *Video S4*). Although risky, rotational atherectomy was unavoidable. We used a 1.5 mm burr for segment 3 and a 2 mm burr for segment 1, but indentation remained. We then applied 40 pulses of 3.5 mm intravascular lithotripsy to each segment and achieved full balloon expansion before stenting both segments. The procedure was successfully completed through single radial access with three stents implanted: 3.5 × 48 mm, 3.0 × 48 mm, and 3.0 × 23 mm. The patient has since remained asymptomatic up to the current 6-month follow-up CT angiogram (*Figure 1H*, see Supplementary material online, *Video S5*).

### Patient 2

**Figure 2:**
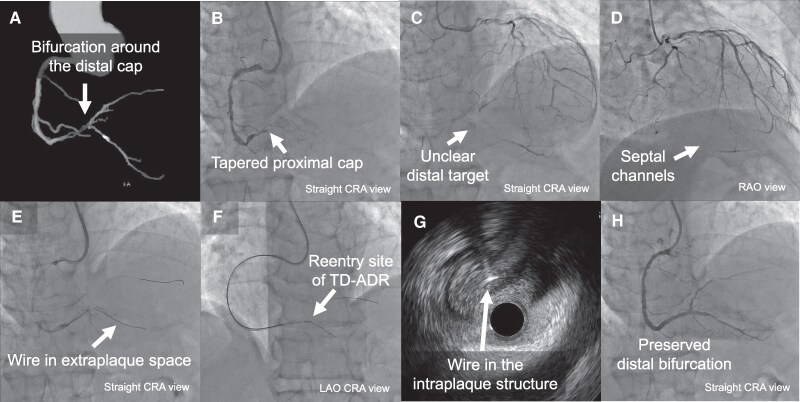
Images of patient 2: (*A*) CT angiogram showing a bifurcation around the distal cap. (*B*) Right coronary angiography demonstrating a tapered proximal cap. (*C*) Left coronary angiography showing an unclear distal target. (*D*) Left coronary angiography showing septal collateral channels. (*E*) A guidewire went extraplaque plane of the posterior descending artery. (*F*) Re-entry site of tip detection-antegrade dissection and re-entry (TD-ADR). (*G*) A Conquest Pro 12ST guidewire in the intraplaque structure. (*H*) Final angiogram showing a preserved distal bifurcation.

A 61-year-old Japanese man with peripheral artery disease presented with Canadian Cardiovascular Society (CCS) Class III effort angina due to an RCA CTO. He had normal renal function and an ejection fraction of 22% with diffuse left ventricular hypokinesis and inferior wall akinesis. Despite optimal medical therapy, he remained symptomatic. Viability testing supported intervention. J-CTO score was 0, PROGRESS CTO score 0. Due to the poor function of the left ventricle, an antegrade-only approach was chosen despite the bifurcation near the distal cap (*Figure 2A–D*, see Supplementary material online, *Videos S6*–*S8*). Via a 7 Fr right radial access using 7 Fr Glidesheath Slender (TERUMO, Japan) and Hyperion (ASAHI INTECC, Japan) 7 Fr AL1 SH guiding catheter, antegrade wiring of the small branch of the PL using a SION black wire (ASAHI INTECC, Japan) was confirmed as intraplaque by IVUS, but PD access failed due to a steep angle. An attempt to reach PD with IVUS guidance led to wiring into an extraplaque plane (*Figure 2E*). Tip detection-antegrade dissection and re-entry in PD was then employed with a Conquest Pro 12ST wire (ASAHI INTECC, Japan), Corsair Pro (ASAHI INTECC, Japan) microcatheter, and OptiCross HD (Boston Scientific, MA, USA) IVUS, successfully entering into the distal true lumen of PD (*Figure 2F* and *G*, see Supplementary material online, *Video S9*).

Subsequent balloon dilations were performed, followed by the implantation of two stents: 3.0 × 48 mm and 2.5 × 38 mm. The procedure concluded with successful recanalization for both branches (*Figure 2H*, see Supplementary material online, *Video S10*). Since the intervention, the patient has been asymptomatic up to the current 7-month follow-up visit.

### Patient 3

**Figure 3:**
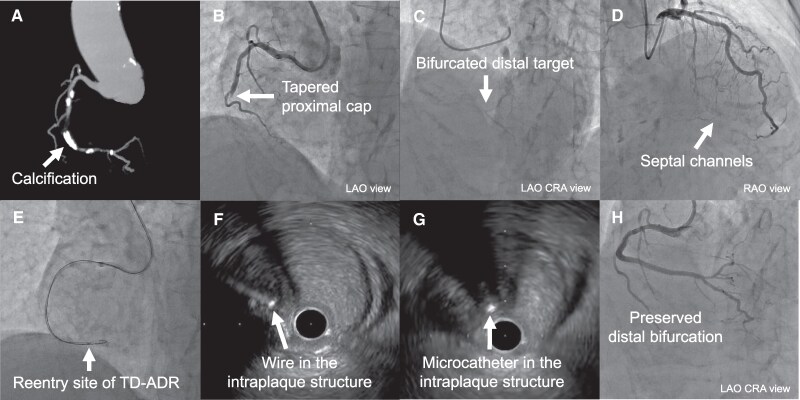
(*A*) CT angiogram highlighting lesion calcification. (*B*) Right coronary angiography demonstrating a tapered proximal cap. (*C*) Left coronary angiography showing a bifurcated distal target. (*D*) Left coronary angiography showing feasible retrograde options. (*E*) Re-entry site of tip detection-antegrade dissection and re-entry (TD-ADR). (*F*) A Conquest Pro 12ST guidewire in the intraplaque structure. (*G*) A microcatheter in the intraplaque structure. (*H*) Final angiogram showing a preserved distal bifurcation.

A 76-year-old Japanese woman with no significant history or risk factors presented with CCS Class III effort angina. Electrocardiogram findings and left ventricular function were normal. Her estimated glomerular filtration rate was 40 mL/min/1.73 m^2^. She underwent PCI for an RCA CTO with a tapered stump and moderate calcification. Diffuse stenotic lesion at the distal target extended close to the bifurcation (*Figure 3A–D*, see Supplementary material online, *Videos S11*–*S13*). J-CTO score was 3, PROGRESS CTO score 0. The plan was a minimalistic approach under the backup of TD-ADR via a single right radial access using a 7 Fr Glidesheath Slender (TERUMO, Japan) and Hyperion (ASAHI INTECC, Japan) 7 Fr AL1 SH guiding catheter. Initial antegrade wiring using a Gladius EX wire (ASAHI INTECC, Japan) failed. We switched to TD-ADR with an AnteOwl IVUS (TERUMO, Japan) and Finecross GT microcatheter (TERUMO, Japan). We punctured the true lumen with a Conquest Pro 12ST wire (ASAHI INTECC, Japan) (*Figure 3E* and *F*). After confirming true lumen entry of the microcatheter, we exchanged for a SION black wire (ASAHI INTECC, Japan) (*Figure 3G*, see Supplementary material online, *Video S14*). Stent implantation was performed using two stents: 3.0 × 16 mm and 2.5 × 48 mm. Final angiography showed excellent results and preserved side branch (*Figure 3H*, see Supplementary material online, *Video S15*). Since the intervention, the patient has been asymptomatic up to the current 9-month follow-up visit.

## Discussion

### The importance of safety in chronic total occlusion percutaneous coronary intervention

The primary indication for CTO PCI is symptom control; therefore, it should avoid putting patients at excessive risk. Percutaneous coronary intervention is an established treatment for chronic coronary artery disease, however, the guidelines for coronary artery revascularization recently downgraded the recommendation for PCI of CTO from Class 2a to 2b.^5^ The use of retrograde approach, routine dual injections, and 8 Fr sheaths may need to be reconsidered in contemporary CTO PCI procedures. The retrograde approach, though effective, is linked to rare but catastrophic complications and is the strongest predictor of in-hospital major adverse cardiac event after CTO PCI.^6,7^ The retrograde approach also increases perforation risk fourfold, highlighting the need for safer alternatives.^8^

In the current case series, the distal side branches were all preserved. This ‘Minimalistic Approach with Tip detection-antegrade dissection and re-entry’ (MAT) has the potential to enhance safety and improve patient comfort in the treatment of CTO.

### Indication for MAT

MAT can be considered when all of the following conditions are met:

1. The proximal CTO cap is accessible.
2. The CTO does not involve diffuse severe calcification.
3. There are no major side branches near the distal cap.
4. More than two physicians experienced in IVUS interpretation are available, along with the necessary equipment and support staff.

**Figure 4:**
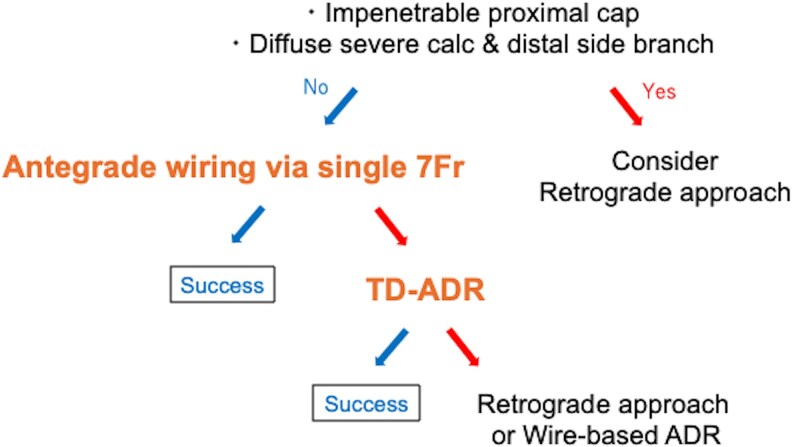
Algorithm for Minimalistic Approach with Tip detection-antegrade dissection and re-entry (MAT) based on lesion characteristics.

Before choosing MAT, it is essential to assess factors beyond anatomy, including operator skill and staffing. MAT may not be feasible when wires cannot penetrate the proximal CTO cap (*Figure 4*, *Table 1*). In diffusely calcified CTOs, TD-ADR is difficult because echo attenuation makes the wire hard to see. Therefore, for CTOs with both a side branch near the distal cap and diffuse calcification, the retrograde approach is still preferred because maintaining PCI success rate should not be sacrificed for minimalistic approach.^9–15^ Coronary CT angiography should be performed beforehand to assess calcium distribution and CTO length.^16^ To compensate for insufficient backup force, since 8 Fr guides are not used, the balloon anchoring technique (in a side branch or main vessel) is often needed, however, the balloon anchoring technique is infeasible in certain situations (i.e. simultaneous IVUS-guided wiring or simultaneous use of a guide-extension catheters with balloon anchoring technique is not possible under 7 Fr guides). Balloon dilation (around 2 mm) of the extraplaque space is often necessary before delivering the IVUS for TD-ADR. Tip detection-antegrade dissection and re-entry does not require the same level of technical sophistication as the retrograde approach, but it does require a thorough understanding of IVUS images, including the directional recognition.

**Table 1:** List of crossing failure cases at Sapporo Cardiovascular Clinic in 2024: failure modes of Minimalistic Approach with Tip detection-antegrade dissection and re-entry (TD-ADR)

	The main reason for crossing failure in minimalistic approach	Vessel	J-CTO score
Failure Case 1	Unable to puncture the proximal cap	LCx	3
Failure Case 2	Due to perforation at the CTO entry	LAD	1
Failure Case 3	Switching to STAR due to difficulties with TD-ADR	RCA	3
Failure Case 4	Due to wire entrapment within the occluded coronary aneurysm	LCx	2
Failure Case 5	No wire can be advanced into the long and calcified CTO	RCA	4
Failure Case 6	Due to perforation at the CTO entry	RCA	3
Failure Case 7	Unable to puncture the proximal cap	LCx	2
Failure Case 8	Due to perforation at the CTO entry	LCx	3

### Difficulty associated with ‘blind wiring’: visualization

**Figure 5:**
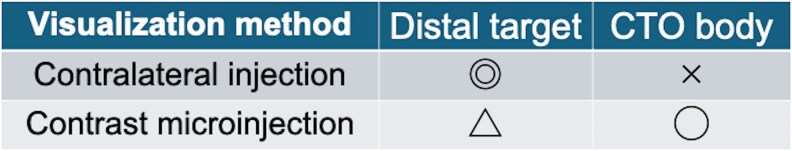
Comparison of contrast microinjection and contralateral injection as visualization methods during chronic total occlusion percutaneous coronary interventions.

One major limitation of MAT is its blind wiring, as it omits simultaneous dual injection and confirmatory contralateral injection. The essential problem with CTOs is that the CTO body is basically invisible, therefore visualization is a key to CTO PCI. We can roughly understand where the wire has advanced using ≤0.5 mL of contrast microinjection, which is an injection with the microcatheter tip in a CTO body like HydroDynamic contrast Recanalization or Carlino technique (*Figure 5*).^17–19^ Gear position can be clarified using a contrast microinjection into the CTO segment, and if the injected contrast unexpectedly expands an extraplaque space, TD-ADR can serve as a bailout.

In cases of long tortuous and calcified CTOs, knuckle wiring might be safer. Prior angiograms should be meticulously reviewed multiple times, thereby minimizing the risk of perforation (especially, perforation in small side branches around distal cap). Hard penetration wires should be avoided and only used in limited situations like when performing an IVUS-guided puncture on an ambiguous proximal cap in MAT.^20^ However, in situations where safety cannot be ensured or the next procedural step cannot be determined without confirming the relative position between the distal target and the wire, we recommend performing a confirmatory contralateral injection without hesitation. This is because the confirmatory contralateral injection itself carries no risk.

### Comparison data for MAT

By using the TD-ADR as a substitute for retrograde approach and the contrast microinjection as a substitute for contralateral injection, it was possible to complete CTO treatment using a single 7 Fr access in most of our cases.

We compared our CTO PCIs in 2024 (i.e. minimalistic era) with those in the previous three years (i.e. retrograde era) when the retrograde approach was still the primary strategy and when we weren’t skilled at TD-ADR yet (*Table 2*). In the minimalistic era, TD-ADR, which was the core tactic, was used in 28% of cases, retrograde approach in 2%, dual access in 7%, 8 Fr in 2%, femoral artery access in 24%. In the minimalistic era, contralateral injection was used only when contrast microinjection is not enough to confirm where the wire had advanced. Between the minimalistic era and the retrograde era, there was no statistically significant difference in the rate of crossing success defined as successful wire passage into the distal true lumen, technical success defined as achieving final TIMI 3 flow with residual stenosis of less than 30%, and procedural success defined as technical success without in-hospital adverse events. In the minimalistic era, CTO procedure time was significantly shorter and the contrast volume was significantly smaller compared to the retrograde era. Some perforations during the minimalistic era were attributable to the lack of contralateral injection; however, the incidence of perforations requiring treatment was 11%, which was not significantly different from that observed in the retrograde era.

**Table 2:** Comparison of chronic total occlusion percutaneous coronary interventions between the minimalistic era (2024) and the retrograde era (2021–23) in Sapporo Cardiovascular Clinic

	Minimalistic era (2024) *n* = 184	Retrograde era (2021–23) *n* = 286	*P*
Femoral approach, *n* (%)	44 (23.9)	235 (82.2)	<0.0001
8 Fr, *n* (%)	4 (2.2)	162 (56.6)	<0.0001
Dual access, *n* (%)	13 (7.1)	163 (57.0)	<0.0001
Retrograde approach, *n* (%)	4 (2.2)	48 (16.8)	<0.0001
Crossing success, *n* (%)	176 (95.7)	271 (94.8)	0.66
Technical success, *n* (%)	170 (92.4)	263 (92.0)	0.86
Procedural success, *n* (%)	168 (91.3)	258 (90.2)	0.69
Procedural time, min	140.2 ± 68.7	166.2 ± 86.2	<0.0001
Fluoroscopy time, min	52.5 ± 33.0	62.4 ± 42.5	0.002
Contrast volume, mL	209.2 ± 68.0	226.7 ± 86.5	0.01
Radiation dose, Gy	2.1 ± 1.4	2.6 ± 1.6	0.002
Insurance-based device cost, yen	682 855 ± 267 107	796 677 ± 306 505	<0.0001
Clinical perforation, *n* (%)	20 (10.9)	32 (11.2)	0.91
Cardiac tamponade, *n* (%)	2 (1.1)	4 (1.4)	0.77

### Future perspective

It is currently difficult to decide whether to choose TD-ADR or retrograde approach due to the lack of practical guidance. Tip detection-antegrade dissection and re-entry needs to be incorporated into CTO crossing algorithms in the future.

## Conclusions

This case series presents CTO treatments performed via single radial access, utilizing the support of the TD-ADR technique. The MAT has the potential to not only reduce procedural risks but also lessen the overall burden on patients, including financial costs.

## Supplementary Material

ytaf355_Supplementary_Data

## Data Availability

The data underlying this article are available in the article and in its online Supplementary material. Further information can be obtained from the following sources: (X) @Laserrman, (Linkedin) Tsutomu Dr Fujita, and (Blog) https://rotamanlaser2.blogspot.com/.

## Lead author biography

Yutaka Tadano was born in 1979 in Hokkaido, Japan. He graduated from Asahikawa Medical University in 2004. Currently, he is the Director of Cardiovascular Medicine at the Sapporo Cardiovascular Clinic in Hokkaido, Japan. His primary areas of expertise and interest include percutaneous coronary intervention for complex, high-risk, and indicated patients, as well as chronic total occlusion.
